# A Clinical Algorithm to Identify HIV Patients at High Risk for Incident Active Tuberculosis: A Prospective 5-Year Cohort Study

**DOI:** 10.1371/journal.pone.0135801

**Published:** 2015-08-17

**Authors:** Susan Shin-Jung Lee, Hsi-Hsun Lin, Hung-Chin Tsai, Ih-Jen Su, Chin-Hui Yang, Hsin-Yun Sun, Chien-Chin Hung, Cheng-Len Sy, Kuan-Sheng Wu, Jui-Kuang Chen, Yao-Shen Chen, Chi-Tai Fang

**Affiliations:** 1 Faculty of Medicine, School of Medicine, National Yang-Ming University, Taipei, Taiwan; 2 Section of Infectious Diseases, Department of Internal Medicine, Kaohsiung Veterans General Hospital, Kaohsiung, Taiwan; 3 Institute of Epidemiology and Preventive Medicine, College of Public Health, National Taiwan University, Taipei, Taiwan; 4 Department of Infection Control and Internal Medicine, E-Da Hospital/I-Shou University, Kaohsiung, Taiwan; 5 Institute of Clinical Medicine, School of Medicine, National Yang-Ming University, Taipei, Taiwan; 6 National Health Research Institute, Zhu-nan, Taiwan; 7 Centers for Disease Control, Ministry of Health and Welfare, Taipei, Taiwan; 8 Division of Infectious Diseases, Department of Internal Medicine, Taipei Medical University Hospital, Taipei Medical University, Taipei, Taiwan; 9 Division of Infectious Diseases, Department of Internal Medicine, National Taiwan University Hospital and College of Medicine, National Taiwan University, Taipei, Taiwan; FIOCRUZ, BRAZIL

## Abstract

**Background:**

Predicting the risk of tuberculosis (TB) in people living with HIV (PLHIV) using a single test is currently not possible. We aimed to develop and validate a clinical algorithm, using baseline CD4 cell counts, HIV viral load (pVL), and interferon-gamma release assay (IGRA), to identify PLHIV who are at high risk for incident active TB in low-to-moderate TB burden settings where highly active antiretroviral therapy (HAART) is routinely provided.

**Materials and Methods:**

A prospective, 5-year, cohort study of adult PLHIV was conducted from 2006 to 2012 in two hospitals in Taiwan. HAART was initiated based on contemporary guidelines (CD4 count < = 350/μL). Cox regression was used to identify the predictors of active TB and to construct the algorithm. The validation cohorts included 1455 HIV-infected individuals from previous published studies. Area under the receiver operating characteristic (ROC) curve was calculated.

**Results:**

Seventeen of 772 participants developed active TB during a median follow-up period of 5.21 years. Baseline CD4 < 350/μL or pVL ≥ 100,000/mL was a predictor of active TB (adjusted HR 4.87, 95% CI 1.49–15.90, *P* = 0.009). A positive baseline IGRA predicted TB in patients with baseline CD4 ≥ 350/μL and pVL < 100,000/mL (adjusted HR 6.09, 95% CI 1.52–24.40, *P* = 0.01). Compared with an IGRA-alone strategy, the algorithm improved the sensitivity from 37.5% to 76.5%, the negative predictive value from 98.5% to 99.2%. Compared with an untargeted strategy, the algorithm spared 468 (60.6%) from unnecessary TB preventive treatment. Area under the ROC curve was 0.692 (95% CI: 0.587–0.798) for the study cohort and 0.792 (95% CI: 0.776–0.808) and 0.766 in the 2 validation cohorts.

**Conclusions:**

A validated algorithm incorporating the baseline CD4 cell count, HIV viral load, and IGRA status can be used to guide targeted TB preventive treatment in PLHIV in low-to-moderate TB burden settings where HAART is routinely provided to all PLHIV. The implementation of this algorithm will avoid unnecessary exposure of low-risk patients to drug toxicity and simultaneously, reduce the burden of universal treatment on the healthcare system.

## Introduction

Tuberculosis (TB) remains the major cause of death in people living with HIV (PLHIV) worldwide [1]. The World Health Organization (WHO) estimates that 13% of the 8.6 million new TB cases in 2012 was co-infected with HIV, causing almost a quarter of the 1.3 million TB-related deaths [2]. HIV infection accelerates progression of latent TB infection into active TB disease [3]. HIV-infected persons have an approximately 30 times increase in the incidence of active TB [4]. While the use of highly active antiretroviral therapy (ART) reduces the risk of TB by 70%–90%, the incidence of TB remains two to four-fold higher than HIV-negative populations [5–9]. An important strategy to further reduce HIV-associated TB is to provide isoniazid preventive therapy (IPT) [8, 10], which has been demonstrated to decrease TB incidence by 27%–37% in patients receiving ART, regardless of the tuberculin skin test (TST) or interferon gamma releasing assay (IGRA) results [11–13].

WHO currently recommends that all PLHIV without evidence of active TB in resource-constrained, high-TB-incidence settings, should be provided with IPT [14]. It remains uncertain, however, whether such recommendations should be extended to HIV-infected positive persons who are not known TB contacts in low-to-moderate TB-burden settings where ART is routinely used to treat all PLHIV. In such settings, untargeted IPT will place large numbers of persons, who may never benefit from IPT, at risk for adverse drug reactions [15]. The preventive treatment would be justifiable only if IPT was targeted to those with significantly increased risk of TB. Nevertheless, no single test available currently can accurately predict the risk of TB in PLHIV.

In HIV patients under profound immunosuppression status, both TST and IGRA may yield false-negative or indeterminate results [3, 16–17]. Studies showed that TST failed to identify 61% (20/33)[12] to 66% (27/41)[13] of PLHIV who develop incident active TB within 4 years of testing and thus may benefit from IPT, and IGRA failed to identify 53% (21/40)[13] of such patients. We hypothesized that the accuracy of predicting risk of TB can be improved by incorporating the patients’ CD4 cell count and HIV viral load as predictors, since both are known markers for HIV-associated immunosuppression that increase the risk of incident active TB; while for those with relatively intact immunity, tests for latent TB infection should still be useful to predict risk of active TB. Whether this algorithm approach can be used to guide targeted IPT for PLHIV has not been previously investigated.

Taiwan is a moderate-TB-burden country (annual TB incidence: 53/100,000 population in 2012)[18], where ART is routinely used to treat all eligible PLHIV since 1997. Taiwan CDC has a policy endorsing the use of IPT in PLHIV, but similar to many countries worldwide, uptake has been limited due to lack of programs to implement the policy [19]. Major concerns against the use of IPT include unnecessary isoniazid hepatotoxicity (8%) [20] when the risk of TB has been greatly reduced by both ART [5] and routine childhood BCG vaccination [21–22], as well as the lack of durable protection of IPT in PLHIV [12–13]. These concerns led to an extremely low uptake of latent TB infection testing and treatment among PLHIV who are not known TB contacts in Taiwan.

This prospective, cohort study aimed to develop and validate a simple, easy-to-use clinical algorithm, based on baseline CD4 cell count, plasma HIV viral load, and IGRA results, to identify those HIV patients who are at an increased risk of active TB and thus are most likely to benefit from IPT in an intermediate TB burden country.

## Methods

### Ethical Statement

Institutional review boards in both hospitals (Institutional Review Board, Kaohsiung Veterans General Hospital and Institutional Review Board, E-Da Hospital) approved the study protocol. All of the participants gave written informed consent.

### Settings

This study was conducted at a medical center, Kaohsiung Veterans General Hospital (Kaohsiung, Taiwan), and a regional hospital, E-Da Hospital (Kaohsiung, Taiwan) in southern Taiwan.

### Study Design

This prospective, cohort study invited all HIV-infected adult patients who attended outpatient clinics at the study hospitals, from Jan, 2006 to Dec, 2010. The primary outcome was incident active TB. None of the participants were involved in TB contact investigations at the time of enrollment. Validation of the algorithm was done using 2 separate, HIV-infected cohorts in Northern Taiwan from previous published studies [23–24].

### Baseline Evaluations

At study entry, clinical evaluation, chest radiograph, and IGRA were performed. Information on demographic data, HIV risk factors, history of BCG vaccination, previous exposure to TB, and past TB disease were obtained by using a standard questionnaire. Previous exposure to TB was considered present if the patient reported ever living with or having contact with a person with active TB in their lifetime. Those with active TB at baseline, as evident by compatible clinical symptoms (such as fever, chills, productive cough, anorexia, body weight loss, etc.), radiologic findings and mycobacterial cultures, were given treatment and not eligible for the study. Those who developed TB within the 60 days run-in period after study entry were assumed to have active TB at baseline and were excluded. Baseline CD4 cell counts and HIV viral loads (tested within three months before study entry), and whether the patients were already receiving ART, were obtained from the medical records. All participants with a CD4 cell count < 350 cells/μL were treated with ART according to contemporary national guidelines [25]. The IGRA results were provided to the physician in charge, and patients testing positive for IGRA were offered the choice to receive IPT or not, after discussion with their primary care physicians. IPT was initiated at the discretion of the in-charge physician.

### IGRA

QuantiFERON-TB GOLD test (Cellestis Ltd, Melbourne, Australia) was performed and interpreted according to the manufacturer’s instructions. The result was positive if the IFN-γ response to TB antigen minus background levels was ≥ 0.35 IU/mL, negative when the IFN-γ response to TB antigen minus background levels was < 0.35 IU/mL, and indeterminate if the background IFN-γ level was ≥ 8.0 IU/mL, or IFN-γ response to phytohaemagglutinin < 0.5 IU/mL.

### Follow Up and Ascertainment of Outcome

All patients were scheduled to be followed up every three months, until they developed active TB disease (event), died from other causes (censored), started IPT (censored) or were censored on Dec 31, 2012. The majority (98.3%) was followed up for a minimum of two years. Because participants may seek medical treatment for active TB in other hospitals, we used the national TB registry to ascertain the outcome during the study period.

### Validation Cohort

Validation of the clinical algorithm was done using 2 separate, HIV-infected cohorts, derived from previously published studies [23–24]. Validation cohort 1 consisted of 912 HIV-infected persons from 3 HIV outpatient clinics and 2 prisons located in Northern Taiwan [23], and validation cohort 2 consisted of 543 HIV-infected patients at the HIV clinic in another medical center in Northern Taiwan, for whom CD4 cell counts, HIV viral load and IGRA results are available [24]. Both cohorts did not receive IPT, and used T-SPOT.TB test (Oxford Immunotec, Oxford, United Kingdom) as the interferon-gamma release assay. T-SPOT.TB also uses ESAT-6 and CFP-10 as the specific antigens for *M*.*tuberculosis* for stimulation of lymphocytes, and uses ELISPOT for enumerating lymphocytes with interferon gamma release. Area under the receiver operating characteristic (ROC) curve was calculated.

### Statistical Analysis

All statistical analyses were conducted using Stata release 10 (STATA Corp, College Station, TX, USA). A *P* value of less than 0.05 was considered significant, and *P* values were two-sided. CD4 cell counts cut-off of 350 cells/μL and an HIV viral load cut-off of 100,000 copies/mL was chosen based on universally accepted cut-offs; CD4 < 350 cells/μL was the cut-off to initiate antiretroviral therapy, and an HIV viral of > 100,000 copies/mL represented a high viral load that may affect antiretroviral treatment response. We used Cox proportional hazards regression model to analyze the predictors for incident active TB. In multivariable models, age and baseline CD4 count was adjusted for, since CD4 count is a known risk factor for IGRA positivity and reactivation of TB. The forward stepwise selection method and likelihood ratio test was used to select the final model. Validation was done by comparing the area under the ROC curve of the 2 separate, HIV-infected cohorts.

## Results

### Participants

**Fig 1:**
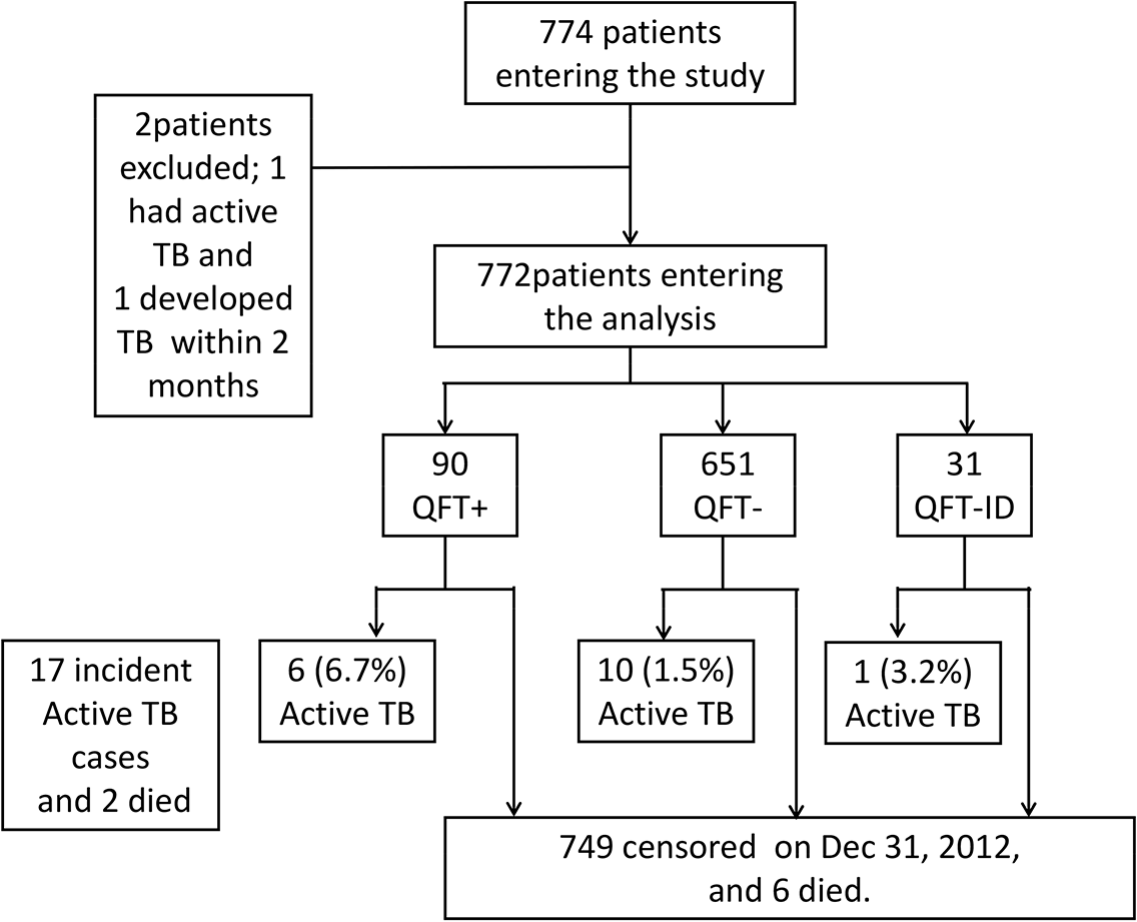
Flowchart of HIV-infected patients who entered the study, and the proportion who developed tuberculosis, stratified by results of interferon-gamma release assay (IGRA).

A total of 774 adult HIV patients agreed to be enrolled. Two were excluded from study (one had active TB disease at entry and the other developed TB within 42 days) (Fig 1). The mean age of the 772 participants was 36.8 years (SD 9.0, range 20.0–77.2), most were men (744, 96.4%). HIV risk factors included injection drug users (IDUs) (517, 67.0%), unprotected sexual transmission (men who have sex with men in 187, 24.2% and heterosexuals in 62, 8.0%) and others (6, 0.8%). Nearly all participants had received BCG vaccination (755, 97.8%) (Table 1).

**Table 1:** Baseline Characteristics of Participants (N = 772).

Characteristics		
Mean age ± SD (years)	36.8 ± 9.0	
(range)	(20.0–77.2)	
Men	744	(96.4)
Median duration of HIV diagnosis (years)	1	(0–3)
Baseline CD4 cell count (cells/μL)		
< 200	66	(8.5)
200–349	161	(20.9)
≥ 350	545	(70.6)
HIV-1 RNA viral load (copies/mL)		
< 10,000	597	(77.3)
10,000–99,999	161	(20.9)
≥ 100,000	14	(1.8)
Log HIV-1 RNA viral load (copies/mL)	3.40	(1.74–3.97)
Use of HAART at entry	254	(32.9)
Use of HAART anytime during the study	478	(61.9)
BCG vaccination	755	(97.8)
Past history of TB	40	(5.2)
Past exposure to TB	35	(4.5)

Data are described as n (%), mean (SD, range) or median (IQR). BCG, Bacille-Calmette Guérin; HAART, highly active antiretroviral therapy; HIV, human immunodeficiency virus; IDU, injection drug users; IQR, interquartile range; MSM, men-who-have-sex-with-men; TB, tuberculosis.

### Baseline CD4 Cell Counts and HIV Viral Load

At study entry, 70.6% (545/772) participants had a CD4 cell count of ≥ 350 cells/μL. Almost a third (254, 32.9%) in overall was receiving ART, and 41% (93/227) with CD4 cell count < 350 cells/μL was on ART. Many patients were newly diagnosed, and were placed on ART thereafter, reaching 68% (154/227) ART coverage for those eligible for ART based on treatment guidelines at the time (i.e. initiation of ART when CD4 cell count < 350 cells/μL).

The median CD4 cell count was 460 cells/μL (IQR 329–634) and the median log_10_ plasma HIV viral load was 3.40 copies/mL (IQR 1.74–3.97) (Table 1). Fourteen patients had HIV viral loads > 100,000 copies/mL, of which ten were newly diagnosed with HIV infection, and four had virological failure.

### Baseline IGRA Results

IGRA was positive in 90 (11.7%) and indeterminate in 31 (4.0%) of subjects. The median CD4 cell count was significantly higher in patients with positive compared with negative IGRA results (511 vs 459 cells/μL, p = 0.01), and significantly lower in those with indeterminate results (339 vs 459 cells/μL, p = 0.01). Patients with low CD4 cell counts of less than 200 cells/μL were less likely to be IGRA positive (5/66, 7.6% vs 85/706, 12.0%, p = 0.02), and had more indeterminate results (7/66, 10.6% vs 24/706, 3.4%, p = 0.01). Factors associated with a positive IGRA on multivariable analysis include age (adjusted OR 1.03, 95% CI 1.00–1.06, p = 0.02), IDU (adjusted OR 2.44, 95% CI 1.31–4.55, p = 0.005), a past history of TB disease (adjusted OR 5.44, 95% CI 2.06–14.36, p = 0.001) and past exposure to TB (adjusted OR 2.77, 95% CI 1.11–6.89, p = 0.03) are shown in Table 2. CD4 cell counts and HIV-1 RNA viral load were not significant. None of the IGRA-positive participants accepted the offer of IPT.

**Table 2:** Factors Associated with a Positive Interferon-Gamma Release Assay (IGRA) Result in 741 Participants*.

Variables	Crude OR	(95% CI)		*P* Value	Adjusted OR^†^	(95% CI)	*P* Value
Age ‡ (years)	1.02	(1.00–1.05)		0.04	1.03	(1.00–1.06)	0.02
Sex, men	0.38	(0.15–0.92)		0.03	039	(0.16–0.99)	0.05
HIV risk factor							
Sexual transmission	1.00				1.00		
IDU	1.71	(1.02–2.89)		0.04	2.44	(1.31–4.55)	0.005
CD4 cell count (cells/μL)							
< 200	0.59	(0.23–1.52)		0.27	0.53	(0.18–1.56)	0.25
200–349	0.59	(0.32–1.11)		0.10	0.55	(0.29–1.05)	0.07
≥ 350	1.00				1.00		
HIV-1 RNA viral load (copies/mL)							
< 10,000	1.00				1.00		
10,000–99,999	0.73	(0.41–1.31)		0.29	0.74	(0.41–1.35)	0.32
≥ 100,000	0.56	(0.07–4.39)		0.58	1.25	(0.13–11.85)	0.84
BCG vaccination	1.04	(0.23–4.61)		0.96	1.26	(0.27–5.79)	0.77
Past history of TB disease	2.78	(1.25–6.17)		0.01	5.44	(2.06–14.36)	0.001
Past exposure to TB	2.78	(1.25–6.17)		0.01	2.77	(1.11–6.89)	0.03

BCG, Bacille-Calmette Guérin; CI, confidence interval; HIV, human immunodeficiency virus; IDU, injection drug users. OR, odds ratio; TB, tuberculosis

*Excluding 31 subjects with indeterminate interferon-gamma release assay result.

†Adjusted for age, sex, HIV risk factor, CD4 cell counts and a past history of TB disease.

‡Test for trend for age: OR 1.03, 95% CI 1.01–1.06, p = 0.02 (adjusted for sex, HIV risk factor, CD4+ T cell count and past TB disease).

### Incidence and Risk Factors of Developing Active TB Disease

**Fig 2:**
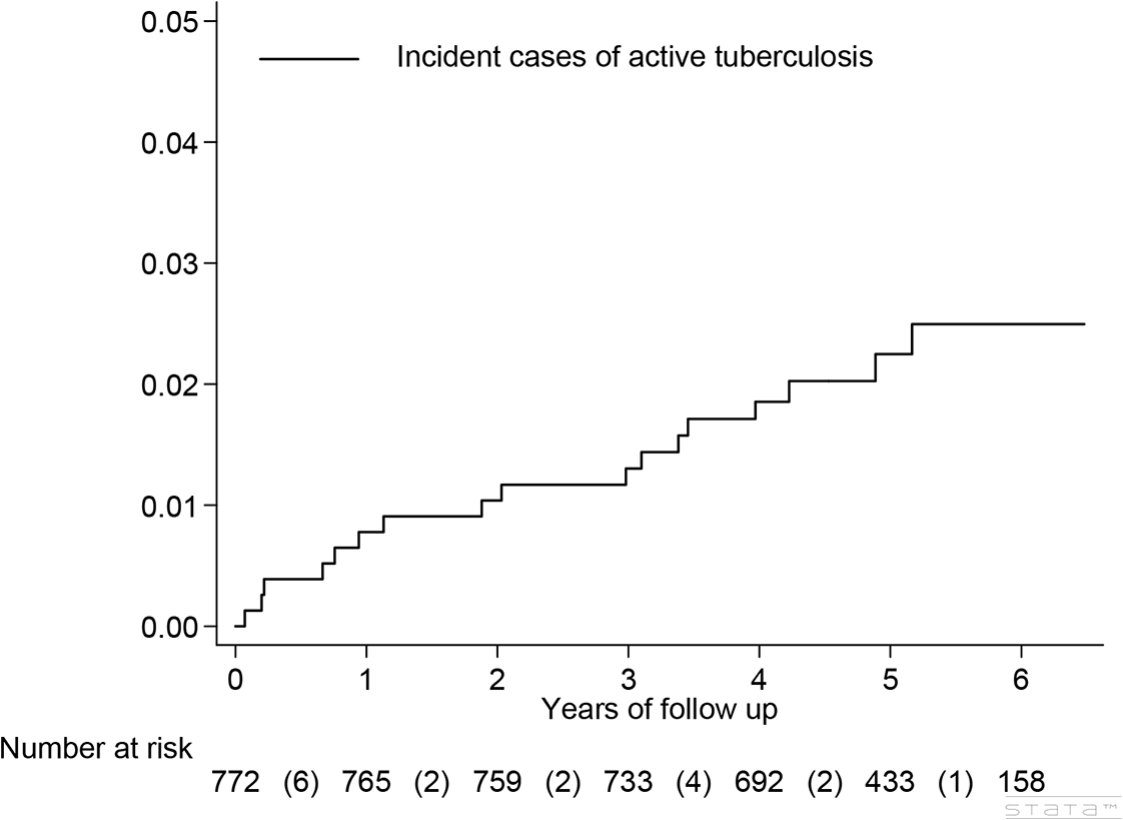
Cumulative incidence of active tuberculosis disease over 5 years in a cohort of HIV-infected adults (N = 772).

Seventeen incident TB cases (2.2%, 17/772) occurred during 3842 person-years of follow up (0.44 per 100 person-years). The median duration of follow-up was 5.21 years (IQR 4.14–5.81) (Fig 2). Five (29.4%) had extrapulmonary TB, including pleuritis (1), peritonitis (1), meningitis (1), cutaneous (1), and disseminated (1). The diagnosis of TB was established by culture in 8 and a positive TB-PCR of the sputum in 2 (10/17, 59%), pathology in 2 (12%) and by typical clinical or radiologic findings in the remaining 5 (29%).

The median CD4 cell count of the 17 active TB cases at diagnosis was 365 cells/μL (IQR 241–640). At study entry, eight (8/17) were not eligible for ART according to guidelines at the time of the study (i.e. CD4 cells ≥350 cells/μL). Of the 9 patients eligible for ART at study entry, 5 were on ART already, 1 was placed on ART after study entry, 1 refused ART, and 2 were lost to follow-up during the study until the onset of TB.

Univariate Cox regression analysis shows that a baseline CD4 cell count of 200–349 cells/μL, < 200 cells/μL, a positive baseline IGRA, and a baseline HIV viral load ≥ 100,000 copies/mL were associated with a 2-fold, 3-fold, 4-fold, and 10-fold increase in risk for incident active TB, respectively (Table 3), though not reaching statistical significance for CD4 cell count. Multivariable Cox regression analysis identified a positive IGRA (adjusted hazard ratio [HR] 5.04, 95% CI 1.76–14.46, p = 0.003) and an HIV viral load ≥ 100,000 copies/mL (adjusted HR 8.75, 95% CI 1.22–62.56, p = 0.03) (Table 3) as independent risk factors for incident active TB, after adjusting for age, CD4 cell count and HIV viral load.

**Table 3:** Incident Rate and Risk Factors for Development of Incident Active Tuberculosis Disease in HIV-Infected Persons Using A Composite Risk Factor of High HIV Viral Load, Low CD4 Cell Count and IGRA Positivity (*n* = 772).

						Model 1			Model 2 (composite risk factor)		
Variables	Incidence rate†	(95% CI)	Crude HR	(95% CI)	P value	Adjusted HR*	(95% CI)	P value	Adjusted HR**	(95% CI)	*P* Value
Age (years)											
< 30	0.20	(0.05–0.79)	1.00			1.00			1.00		
30–39	0.48	(0.24–0.96)	2.40	(0.51–11.28)	0.27	2.41	(0.51–11.36)	0.27	2.28	(0.48–10.76)	0.30
≥ 40	0.61	(0.29–1.27)	3.02	(0.63–14.52)	0.17	3.12	(0.64–15.21)	0.16	2.54	(0.53–12.27)	0.25
Sex											
male	0.46	(0.28–0.74)	1.00			—			—		
Female	0.00		—			—			—		
HIV risk factor											
MSM	0.61	(0.25–1.47)	1.00			1.00			1.00		
Heterosexual	0.73	(0.18–2.91)	1.19	(0.23–6.13)	0.84	1.10	(0.21–5.78)	0.91	1.29	(0.25–6.67)	0.76
IDU	0.37	(0.20–0.68)	0∙63	(0.22–1.86)	0.41	0.78	(0.23–2.62)	0.68	0.73	(0.24–2.20)	0.57
BCG vaccination:											
No	0.00		—			—			—		
Yes	0.45	(0.28–0.73)	—			—			—		
Past history of TB:											
No	0.44	(0.27–0.71)	1.00			1.00			1.00		
Yes	0.58	(0.08–4.10)	1.28	(0.17–9.63)	0.81	1.00	(0.12–8.30)	1.00	0.82	(0.11–6.24)	
Past exposure to TB:											
No	0.43	(0.26–0.70)	1.00			1.00			1.00		
Yes	0.75	(0.11–5.32)	1.66	(0.22–12.59)	0.63	1.24	(0.16–9.72)	0.84	1.19	(0.16–9.06)	
CD4 cell counts (cells/μl)											
< 200	1.05	(0.34–3.27)	3.18	(0.86–11.79)	0.08	1.46	(0.27–7.79)	0.66	—		
200–349	0.66	(0.27–1.59)	2.03	(0.68–6.06)	0.21	1.78	(0.59–5.39)	0.31	—		
≥ 350	0.32	(0.17–0.62)	1.00			1.00			—		
HIV-1 RNA viral load (copies/ml)											
< 10,000	0.34	(0.18–0.63)	1.00			1.00			—		
10,000–99,999	0.60	(0.25–1.44)	1.79	(0.61–5.23)	0.29	1.70	(0.58–5.01)	0.34	—		
≥ 100,000	3.43	(0.86–13.73)	9.87	(2.15–45.29)	0.003	8.75	(1.22–62.56)	0.03	—		
Interferon-gamma release assay (IGRA)											
Positive	1.30	(0.58–2.89)	4.18	(1.52–11.52)	0.006	5.04	(1.76–14.46)	0.003	—		
Indeterminate	0.69	(0.10–4.89)	2.16	(0.28–16.87)	0.46	1.66	(0.21–13.38)	0.63	—		
Negative	0.31	(0.17–0.57)	1.00			1.00			—		
**Composite risk factor**											
pVL < 100,000 copies/mL, CD4 ≥ 350 cells/μL, andIGRA negative or indeterminate	0.17	(0.06–0.44)	1.00			—			1.00		
pVL < 100,000 copies/mL, CD4 ≥ 350 cells/μL, and IGRA positive	1.05	(0.39–2.79)	6.28	(1.57–25.13)	0.01	—			6.09	(1.52–24.40)	0.01
pVL ≥ 100,000 copies/mL or CD4 < 350 cells/μL	0.86	(0.45–1.66)	5.10	(0.57–16.60)	0.01	—			4.87	(1.49–15.90)	0.01

BCG, Bacille-Calmette Guérin; CI, confidence interval; HR, hazard ratio; HIV, human immunodeficiency virus; IDU, injection drug users; IGRA, interferon-gamma release assay; MSM, men-who-have-sex-with-men; TB, tuberculosis; pVL, plasma HIV-1 RNA viral load.

*Model 1: Adjusted for age, CD4 cell counts and HIV viral load

**Model 2: Adjusted for age and the composite risk factor of CD4 cell count, HIV viral load, or QuantiFERON.

† per 100 person-years

### Sensitivity and Predictive Values of IGRA

**Fig 3:**
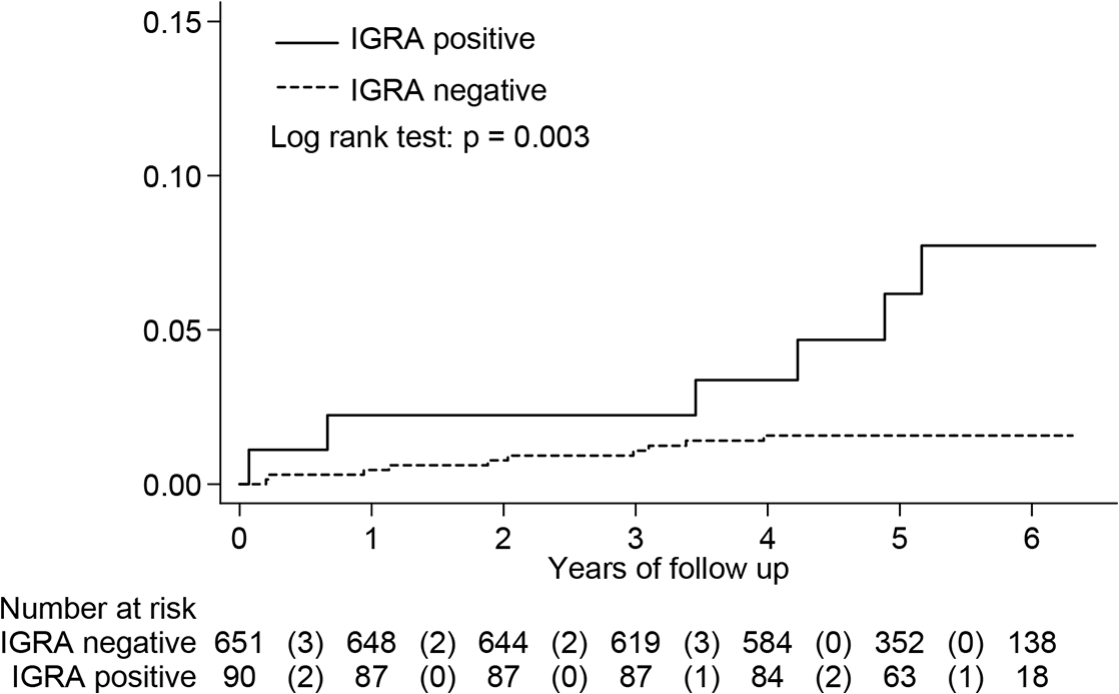
Kaplan-Meier curve of HIV-infected patients who developed active tuberculosis disease by interferon-gamma release assay (IGRA) status, excluding 31 subjects with indeterminate results (N = 741).

HIV-infected patients with a positive IGRA had a cumulative risk for developing active TB of 6.7% (6/90), significantly higher than the 1.5% (10/651) of those with a negative IGRA (log rank test, p = 0.003) (Fig 3). The negative predictive value was 98.5% (641/651). However, IGRA alone identified only 37.5% (6/17) of patients who developed incident active TB during follow-up.

### Algorithm to Predict Risk of Active TB in HIV-Infected Persons

**Fig 4:**
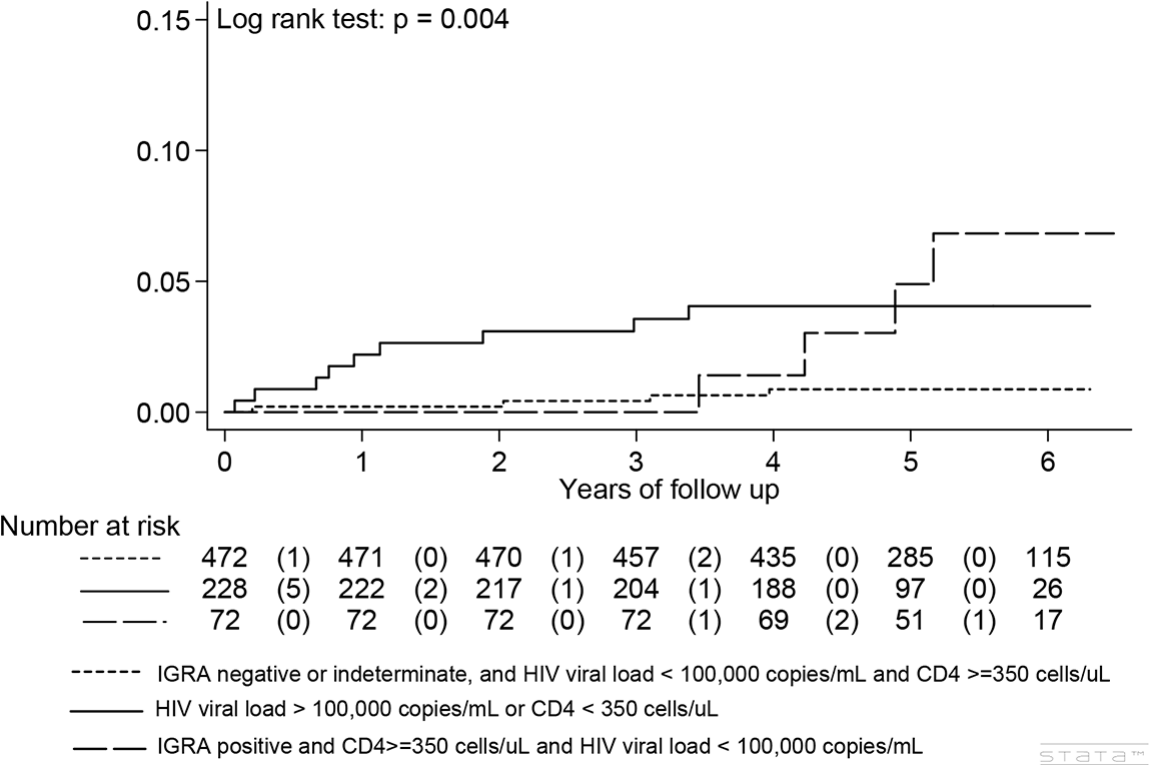
Kaplan-Meier curve to development of active tuberculosis disease by composite risk factor combining high HIV viral load of greater than 100,000 copies per mL, CD4 cell count and interferon-gamma release assay (IGRA)(N = 772).

We constructed composite risk factors using baseline CD4 cell count, plasma HIV viral load, and IGRA results. A composite factor of high HIV viral loads ≥ 100,000 copies/mL or low CD4 cell counts of < 350 cells/μL (adjusted HR 4.7, 95% CI 1.49–15.90, p = 0.009) and a positive IGRA in those lacking the composite factor (adjusted HR 6.09, 95% CI 1.52–24.40, p = 0.01) were two independent risk factors for developing incident active TB (Table 3 and Fig 4).

**Fig 5:**
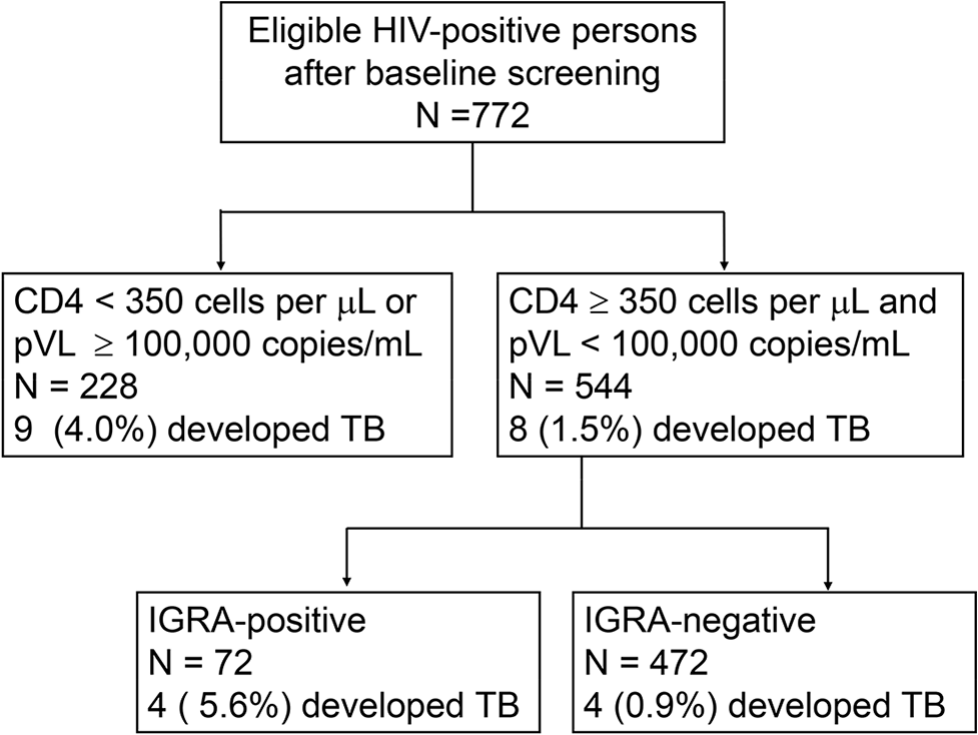
A multivariable algorithm to predict risk of active tuberculosis in people with HIV infection using a composite risk factor of HIV viral load, CD4 cell count and interferon-gamma release assay (IGRA) result.

Based on the above findings, we developed a multivariable clinical algorithm (Fig 5): (1) HIV-infected persons with a CD4 cell count < 350 cells/μL or an HIV viral load ≥ 100,000 copies/mL are at a high risk of developing active TB early (pVL/CD4 approach), and should be offered IPT; (2) those without these two risk factors, but tested positive for IGRA should also be prioritized for IPT (pVL/CD4 approach augmented by IGRA).

### Comparison of Study Algorithm with Alternative Approaches

Compared with an untargeted IPT strategy (WHO approach), the study algorithm targets only HIV-infected persons at high risk for developing incident TB (38.9%, 300/772), and thus avoids unnecessary IPT in 60.6% (468/772) (Table 3). The number needed to treat (NNT) to prevent one case of active TB is reduced from 45 to 23 (Table 4).

**Table 4:** Comparison of Different Approaches to Prevent Tuberculosis in People Living With HIV.

	Study Algorithm*(N = 772)		IGRA-alone Approach*	WHO Approach*
Variable	pVL/CD4 approach†	pVL/CD4 approach augmented by IGRA**	(N = 772)	(N = 772)
Isoniazid preventive treatment needed	228 (29.5)	300 (38.9)	90 (11.7)	772 (100.0)
IGRA testing needed	0	544	772	0
HIV viral load needed	772	772	0	0
CD4 cell count needed	772	772	0	0
False-negative results	8 (1.5)	4 (0.8)	11 (1.6)	0 (0.0)
True positives (Sensitivity)	9 (52.9)	13 (76.5)	6 (35.3)	17 (100.0)
Patient unnecessarily treated	219 (28.4)	287 (31.2)	84 (10.9)	755 (97.8)
Number needed to treat (NNT) (95% CI)assuming 90% efficacy of IPT	25 (14–69)	23 (14–47)	15 (7–81)	45 (29–81)
Number needed to treat (NNT) (95% CI)assuming 50% efficacy of IPT	51 (39–71)	46 (37–61)	30 (21–54)	91 (79–108)
**Characteristics of patients with true positive results selected for treatment**				
No of patients	9	13	6	17
Median CD4 cell count at presentation (cells/μl)	272 (39–441)	287 (39–932)	639 (76–932)	365 (39–932)
Median months to onset of tuberculosis	13.3 (2.8–42.5)	24.5 (2.8–64.0)	48.1 (2.8–64.0)	26.3 (2.8–64.0)
**Characteristics of patients with false-negative results**				
No of patients	8	4	10	—
Median CD4 cell count at presentation (cells/μl)	639 (365–932)	462 (365–902)	337 (39–902)	—
Median months to onset of tuberculosis	46.5 (4.4–64.0)	32.8 (4.4–49.6)	24.5 (4.4–49.6)	—

Data are described as n (%) or median (range), unless otherwise specified. CI, confidence interval. IGRA; interferon-gamma release assay; IQR, interquartile range; pVL, HIV plasma viral load; WHO, World Health Organization.

* pVL/CD4 approach include those with high HIV viral load of > = 100,000 copies/mL and low CD4 cell counts of < 350 cells/μL

**pVL/CD4 approach augmented by IGRA includes those in the pVL/CD4 approach and additionally those with a positive IGRA test; IGRA approach include those with positive IGRA tests only and the W.H.O. approach does no testing and includes all.

†In those with CD4 ≥ 350 cells/μL, the proportion testing IGRA-positive was 72/545 (13.2%), and in those with high HIV plasma viral load ≥ 100,000 copies/mL, 1/14 (7.1%).

Compared with the IGRA-alone strategy, the study algorithm improves the sensitivity to predict the occurrence of incident active TB from 37.5% (6/17) to 76.5% (13/17), and the negative predictive value from 98.5% (641/651) to 99.2% (468/472).

### Validation of the Study Algorithm

Active TB developed in 4 of 912 HIV-infected patients in validation cohort 1 (0.17 per 100 person-years) with 40.6% on antiretroviral therapy at study entry [23]and in one of 543 HIV-infected patients in validation cohort 2 (0.60 per 100 person-years) with 90.5% on ART [24]. The area under the ROC curve was 0.692 (95% CI: 0.587–0.798) in our study cohort and 0.792 (95% CI: 0.776–0.808) in the validation cohort 1 and 0.766 in validation cohort 2, respectively. Active TB developed in 2/309 (0.7%) of HIV-infected individuals with low CD4 cell count (< 350/μL) and high HIV viral load (> = 100,000 copies/mL) in cohort 1. For those with high CD4 cell counts (> = 350 cells/μL) and low HIV viral load (< 100,000 copies/mL), active TB developed in 2/72 (2.8%) and 1/45 (2.2%) of HIV-infected individuals testing positive for T-SPOT.TB, in cohort 1 and 2, respectively. None of the patients with negative or indeterminate results developed active TB in the low risk category with high CD4 cell counts and low HIV viral loads.

## Discussion

Our results demonstrate that a validated, clinical algorithm can identify those HIV patients at high risk for developing active TB. First, those with a CD4 cell count < 350 cells/μL or an HIV viral load ≥ 100,000 copies/mL are at high risk of TB, and should be offered IPT regardless of IGRA results. Second, for those with a CD4 cell count ≥ 350 cells/μL and an HIV viral load < 100,000 copies/mL, IGRA can identify those at high risk of TB who may benefit from IPT. The accuracy of predicting risk of TB is markedly improved by incorporating CD4 cell counts and HIV viral loads into the algorithm. Compared with an IGRA alone approach, the sensitivity was improved from 37.5% to 76.5%; the negative predictive value was improved from 98.5% to 99.2%. Targeting IPT on the high risk group identified by this algorithm will spare 60.6% of the HIV patients from unnecessary treatment compared to untargeted strategy.

The study algorithm was validated in 2 separate, HIV-infected cohorts using T-SPOT.TB as IGRA, from Northern Taiwan [23–24]. Validation using these cohorts found a greater area under the ROC than the study algorithm. Similar to our study (17, 2.2%), a low number of cases of active TB (5, 0.6% and 1, 1.6%) was observed in both validation cohorts. The overall incidence of active TB was similar in our study cohort (0.44 per 100 person-years) compared to the validation cohorts, with an overall incidence rate of 0.17 per 100 person-years [23] in cohort 1 and 0.60 per 100 person-years in cohort 2 [24]. Our results and algorithm validates previous study cohorts, predicting a higher risk of active TB in those with low CD4 cell counts and high HIV viral loads (2/309, 0.7%), and in those with high CD4 cell counts and low HIV viral loads, only when IGRA was positive (2/72, 2.8% and 1/45, 2.2% in cohort 1 and 2, respectively).

A recent, randomized controlled trial in South Africa, a high TB burden country, showed that isoniazid plus ART was effective in preventing TB, and the effect was not restricted to those with positive TST or IGRA, which led to the recommendation for universal IPT in HIV patients receiving ART [13]. However, the participants of this trial had a median CD4 cell count of 216 cells/μL (range: 152–360). The majority of these patients would be categorized as high TB risk by our clinical algorithm, and benefit from IPT. Whether HIV patients with a low-TB risk defined by our algorithm (i.e. those who had a CD4 cell count ≥ 350 cells/μL, an HIV viral load < 100,000 copies/mL, and a negative IGRA), can benefit from IPT can only be answered by future studies.

Our finding that a composite risk factor of either a low baseline CD4 cell count of < 350 cells/μL or a high HIV viral load ≥ 100,000 copies/mL as an independent predictor for incident active TB in HIV persons (adjusted HR 4.87) is consistent with existing literature. Previous studies showed that the risk of incident active TB in untreated HIV-infected persons increases to more than 4-fold of the baseline risk when the CD4 cell counts decrease to less than 350 cells/μL [26]. In those receiving HAART, the risk of incident active TB was associated with a low baseline CD4 cell count [2, 6, 27]. In addition to CD4 cell counts of less than 350, a high viral load of > 10,000 copies/mL was shown to be one of the risk factors associated with TB developing after more than six months of HAART [27]. Similarly, this study shows that HIV-infected people with high viral loads of ≥ 100,000 copies/mL are at high risks of developing active TB early, regardless of CD4 cell counts.

Previous studies have shown that false negative and indeterminate results on IGRA in HIV-infected persons are associated with low CD4 cell counts, as was seen in our analysis [16, 28]. In our study, 31 patients (4%) had an indeterminate IGRA result, a group with significantly lower CD4 cell counts than those with either a positive or negative IGRA. However, a positive IGRA test in HIV-infected persons with relatively intact immunity (CD4 cell count > 350 cells/μL and HIV viral load < 100,000 copies/mL) can improve the estimation for the risk of incident active TB, and increases the sensitivity from 52.9% (HIV viral load and CD4 approach alone) to 76.5% (HIV viral load and CD4 approach augmented with IGRA).

The positive predictive value (6.5%) and negative predictive value (98.5%) of IGRA for active TB in our study are similar to previously reported data [29–35]. A recent meta-analysis found IGRA to have a pooled positive predictive value of 8.5% (55/648) in healthy persons in contact with active TB cases, and 6.0% (11/182) in 4 HIV studies [31]; but only two of these studies were designed to address the ability of IGRA to identify HIV-infected individuals likely to progress to incident active TB [35–36]. The first study was conducted in Austria, a country with low TB incidence, and found that 8.3% (3/36) of HIV-infected persons with a positive IGRA developed TB disease within 19 months of follow up, with a negative predictive value of 100% (738/738)[35]. The second study, conducted in Kenya, a high TB incidence country, which found that IGRA had a positive predictive value of 5.5% (6/110) among HIV-infected pregnant women during a mean follow up of 1.28 years, with a negative predictive value of 98.0% (145/148)[36].

The overall incidence of active TB in our study (2.2%, 0.44 per 100 person-years) was higher than that observed in Austria, a low TB transmission country (0.4%),[35] but much lower than the 2.7 per 100 person-years observed in Kenya, a high TB transmission country [36]. The incidence of active TB in those with a positive IGRA in the present study (1.30 per 100 person-years) was 70% lower than that observed in Kenya (4.2 per 100 person-years)[36]. The difference in active TB incidence is most likely explained by a difference in ART coverage between the two countries. The free provision of ART to all HIV-infected patients in Taiwan is an important protecting factor against TB in the present study.

None of the participants tested IGRA positive chose to receive IPT. Taiwan CDC has a policy endorsing the use of IPT in HIV-infected patients, but similar to many countries worldwide[19], uptake has been limited due to lack of programs to implement the policy [24], perceived low risk of active TB [24], and concerns with INH hepatoxicity, which has an overall case-fatality rate of 10% in those with clinically apparent hepatitis. Tuberculin skin test is not provided for testing to adults due to high coverage of neonatal BCG vaccination in Taiwan since 1965, with booster doses given to school children until 1997 [23]. Currently in Taiwan, IPT is only offered to children under contact investigations since 2008 and to young adults born before 1987, as of April 2012 [22].

IGRA is not readily available and is not reimbursed by the national health insurance for testing. This led to an extremely low uptake of latent TB infection testing and treatment among HIV patients in Taiwan, which, was accepted as the standard of care during the study period (2006–2010) in Taiwan. The free provision of ART for HIV-infected patients eligible for treatment provided very effective protection against TB. The additional protective effect of IPT in HIV-infected patients on ART was suggested by retrospective cohort studies [19, 29, 37] and was not established until 2014, when a randomized control trial of isoniazid plus ART to prevent TB in high TB burden country was published [13]. The risk of active TB is 11 times higher than the general population, even in a cohort with 90% ART coverage [24]. Our clinical algorithm may provide a tool for programs to implement policy, and facilitate increase uptake of IPT in addition to ART in countries with intermediate TB burden.

TSPOT.TB uses the same antigens specific for QuantiFERON-TB Gold test used in this study (ESAT-6 and CFP-10), thus theoretically the results of this study could be generalizable to T-SPOT.TB. Although studies comparing the 2 IGRAs (T-SPOT.TB and QFT) in HIV-infected individuals have shown concordance which varied between poor (kappa = 0.15)[38] to fair (kappa = 0.35)[39] to moderate (kappa = 0.56)[40], validation of this clinical algorithm in 2 separate HIV-infected cohorts in Northern Taiwan using T-SPOT.TB suggests that the conclusions of this study can be extended to T-SPOT.TB.

Our study has several limitations. Firstly, the routine provision of free HAART to all participants with CD4 cell counts of less than 350 cells/μL, according to treatment guidelines, precluded the ability of our study to demonstrate the protective effect of HAART on reducing the risk of incident active TB, due to lack of a comparison group who did not receive HAART. Secondly, we did not evaluate the potential usefulness of TST when combined with CD4 cell count and HIV viral load in stratifying risk for active TB, because cutaneous anergy or false negative TSTs may occur in up to 60% of HIV-infected patients [16–17], and routine BCG vaccination in Taiwan renders TST difficult to interpret due to false positivity. However, TST was shown to be more specific than IGRA in a recent study done in college students with low or no risk for TB [41], and may be incorporated into an algorithm approach in settings where BCG vaccination is not routinely given. Thirdly, generalizability to females is cautioned as almost all study participants were male. Finally, this study was conducted in an economically developed country of moderate TB incidence, where HAART and advanced medical care, including CD4 cell counts, HIV viral loads and IGRA, are available for HIV-infected patients. Our findings therefore may not be generalizable to those settings where medical resources are constrained or with very high TB burdens.

In conclusion, a clinical algorithm incorporating CD4 cell count, HIV viral load, and IGRA status can identify those HIV patients at high risk of incident active TB who are most likely to benefit from IPT. This algorithm can be used to guide targeted TB preventive treatment in PLHIV living in low-to-moderate TB burden settings, while avoiding unnecessary exposure of low-risk patients to drug toxicity and reduce the burden on the healthcare system.

## Supporting Information

S1 Dataset (XLS)
